# Automated emotion recognition via video-based semantic embeddings

**DOI:** 10.3389/fdgth.2026.1808411

**Published:** 2026-05-29

**Authors:** Hannes Diemerling, Patricia Kulla, Joachim Kruse, Timo von Oertzen

**Affiliations:** 1 Faculty of Health, Health and Medical University, Erfurt, Germany; 2 Department of Psychology, Humboldt-Universität zu Berlin, Germany; 3 Thomas Bayes Institute, Berlin, Germany; 4 Department of Psychology, University of the Bundeswehr München, Munich, Germany

**Keywords:** automated facial expression analysis, embedding, emotion recognition (ER), machine learning, transformer

## Abstract

**Introduction:**

Automated emotion recognition systems often rely on acted datasets and categorical models that miss the nuance of spontaneous affect.

**Methods:**

This work assembled a large corpus of authentic facial emotion expressions from naturalistic outpatient psychotherapy sessions, annotated with free-text descriptions by human labelers. These descriptions were embedded in a 768-dimensional semantic space using a fine-tuned German Sentence-BERT model. Transformer, BILSTM, and deep neural network architectures were trained to map facial landmark features to continuous emotion embeddings.

**Results:**

Leave-one-out cross-validation showed model predictions closely matched human annotations with a mean z-score of 1.97. External evaluation against acted datasets (RAVDESS) confirmed strong recognition of joy, sadness, and fear.

**Discussion:**

To enhance interpretability, a back-translation mechanism using cosine similarity was implemented and visualized with radar charts. All components were integrated into AFFECT, an open-source pipeline for analyzing emotional expressions in everyday video recordings.

## 1 Introduction

In clinical practice, particularly in psychotherapy, accurately reading a patient’s emotional state is essential for guiding treatment decisions. Yet this process remains inherently subjective, shaped by clinician experience, and difficult to standardize [1]. Understanding why emotion recognition is so challenging, and how it might be supported by automated tools, requires a closer look at the nature of emotions themselves.

Modern emotion theories describe emotions as complex patterns showing how people judge situations. These patterns involve changes in body functions, brain activity, actions, and personal feelings [2]. Emotions play key roles in thinking, deciding, and socializing. Brain research links these processes to areas like the amygdala, insula, and parts of the prefrontal and temporal lobes. These networks quickly spot emotional cues and help us adapt to our surroundings [3]. From an evolutionary view, emotions let us share vital signals about danger, social needs, and satisfaction or displeasure with others [4, 5].

Given these evolutionary foundations, humans demonstrate remarkable proficiency in emotion recognition. Most individuals can identify emotions from basic facial or vocal expressions within approximately 600 milliseconds, with recognition accuracy for fundamental emotions typically ranging between 60% and 80% across diverse cultural contexts [6, 7]. When describing emotional states, humans frequently reference a core set of basic emotions. Ekman and Friesen [8] systematically characterized these fundamental emotions—fear, anger, disgust, surprise, sadness, and joy—developing a taxonomic system that conceptualizes human emotions as combinations of these elemental components. This taxonomy remains influential due to its biological grounding and cross-cultural applicability, and represents an early, minimalist approach to formal emotion classification.

However, human emotion recognition is highly context-dependent. For instance, a smile may signal genuine happiness in positive social contexts but merely represent social politeness following awkward interactions [9]. Cultural norms further shape emotional expression and interpretation, explaining the in-group advantage whereby individuals recognize emotions more accurately from culturally similar others [10, 11]. Personal characteristics such as empathy, emotional intelligence, and current mood also influence how emotional signals are perceived [6]. Despite its intuitive nature, human emotion recognition is therefore a fundamentally subjective process shaped by multiple interacting factors.

These complexities suggest the value of enriching emotion models beyond categorical approaches. Dimensional frameworks, such as Russell’s circumplex model, replace discrete categories with continuous dimensions—most commonly valence (how pleasant an emotion feels) and arousal (how activating it is)—allowing emotional states to be represented as points in a continuous space rather than as fixed labels [12]. Building on this idea, Barrett’s conceptual-act framework treats valence and activation as building blocks from which specific emotion concepts emerge [13], while appraisal-based models add further dimensions such as potency and novelty to capture a broader range of emotions [14]. These dimensional approaches provide richer emotional representations than categorical models, and their combination of formal rigor and intuitive accessibility makes them attractive for both theoretical research and automated emotion detection.

Automated emotion recognition has become a central research topic spanning affective computing, artificial intelligence, and human-computer interaction, with methods developed for facial expressions, speech, text, and physiological signals. Traditional systems rely on hand-crafted features, such as pitch, energy, and facial landmarks, paired with classifiers like Support Vector Machines or Random Forests [15]. More recently, deep learning approaches using convolutional, recurrent, and transformer-based architectures have enabled end-to-end learning directly from raw data, often outperforming traditional methods across multiple modalities [16, 17]. In clinical settings such as psychotherapy, such tools could complement therapist judgment by providing continuous, objective indices of patient affect throughout treatment.

Emotion recognition works fundamentally differently in humans and machines. Humans experience emotions as multi-component episodes involving subjective feelings, physiological reactions, cognitive evaluations, and expressive behaviors [2], and can only communicate these experiences when they have linguistic labels that capture the relevant distinctions [18]. Machines, by contrast, do not feel emotions; they infer them from observable data such as images, audio, or text, mapping these signals into predefined representational spaces. Most systems either classify emotions into discrete categories based on Ekman’s model [8] or describe them along continuous dimensions like valence and arousal [12]. On certain benchmark datasets, computer vision systems can even outperform humans, achieving around 73% accuracy in distinguishing genuine from posed facial expressions, while human performance remains near chance levels [19].

Despite their individual strengths, no single framework fully captures the complexity of human emotions. Categorical models [8] are intuitive and widely understood but cannot represent blended or subtle affective states [20]. Dimensional models offer continuous representations yet may conflate distinct experiences mapped to similar coordinates [12, 13]. Even richer appraisal-based models compress context-sensitive phenomena into a small set of predefined dimensions [14]. Similarly, feature-engineered systems prioritize interpretability but may miss deeper patterns in affective data, while end-to-end neural models capture complex structures yet require extensive labeled data and can be difficult to interpret [21, 22]. Empirical evidence further shows that social context systematically influences emotion judgments, underscoring the need for more flexible, data-driven approaches that go beyond rigid predefined structures [23].

Natural language processing offers such a framework through semantic embeddings—representations that encode words and sentences as points in continuous vector spaces. Models such as Word2Vec and GloVe learn these representations from large text corpora, positioning semantically related words close together so that geometric relationships reflect meaning relationships [24, 25]. Because distances vary smoothly, the same framework can represent subtle meaning differences without forcing data into fixed categories. For clinical applications, this means that a nuanced description such as “the patient appeared anxious but was trying to maintain composure” can be preserved in its full complexity rather than reduced to a single label like “anxiety.”

Contextual models like BERT extend this idea by generating unique vectors for each word depending on its surrounding context—for example, positioning the word “cold” differently in “cold weather” vs. “cold shoulder” [26]. These models transfer well across tasks and can be fine-tuned with modest data, making them practical tools for handling spontaneous, naturalistic language.

However, current emotion recognition research remains constrained by dependence on acted datasets including the Ryerson Audio Visual Database of Emotional Speech and Song [RAVDESS; [27]] and the Berlin Database of Emotional Speech [EMO-DB; [28]]. While these datasets achieve high production quality, they cannot capture real spontaneous characteristics of non-acted situations, a critical limitation for clinical applications, where emotional expressions are typically subtle, spontaneous, and embedded in complex interpersonal dynamics.

To address this limitation, the current study introduces a novel dataset of authentic emotional expressions from naturalistic psychotherapy sessions, annotated with free-text descriptions rather than categorical labels. These descriptions are embedded in a continuous vector space, enabling the encoding of subtle affective nuances [24, 25]. We train models to map video input to high-dimensional emotion embeddings and implement a back-translation procedure that converts these embeddings back into human-readable descriptors via cosine similarity [24, 29], making the output both nuanced and interpretable for clinicians and researchers alike. The complete system, termed AFFECT (Automated Fast Facial Emotion Coding Tool), is available at https://github.com/HannesDiemerling/AFFECT.

In the following sections, we explain how we created our emotion video database and developed a flexible, data-driven model for emotion recognition. We describe how we tested the model, both with our own data and with established databases. The results section shares the main features of our video database and the outcomes of these tests. Finally, we discuss what our findings mean and why they matter.

## 2 Methods

This section describes the comprehensive pipeline for creating AFFECT, encompassing raw video collection, preprocessing, human annotation, and neural network training to generate continuous affective embeddings from video material. By utilizing naturalistic therapy interactions and applying different processing and labeling procedures, we ensure that the resulting corpus accurately reflects subtle, context-rich emotional expressions suitable for creating an authentic emotion dataset.

### 2.1 Data acquisition

The video material was obtained from outpatient psychotherapy sessions at the Outpatient Clinic of the University of the Bundeswehr in Munich. Each session was recorded using a high-definition camera (1280×720 pixels; 24.97 fps) positioned to capture the whole therapy interaction. All participants (N=20, German native speakers, 12 males, 8 females, age range 22–64 years, mean age = 41.2, SD=12.7) provided written informed consent for video capture and subsequent research use. Ethical approval has been granted by the institutional review board of the University of the Bundeswehr in Munich.

Each recorded session lasted approximately 55 min. To ensure data quality and consistency, we utilized the central 45 min of each session, excluding arrival and departure procedures that might contain non-therapeutic interactions or technical adjustments.

### 2.2 Data creation and video preprocessing

Videos underwent systematic preprocessing to enable and streamline the human annotation process. First, we isolated patient facial expressions from full-room recordings using MediaPipe’s Face Mesh model [30] to identify all visible facial regions within the video material. Video clips achieving less than 50% model confidence in face recognition were excluded from further analysis.

The midpoint coordinates of each detected face were recorded throughout each session, generating spatial coordinate clouds over time. These coordinates were clustered into two distinct groups corresponding to patient and therapist faces using k-means clustering as implemented in the scikit-learn Python library [31, 32].

Building on this patient-therapist separation, we sampled 15-frame clips (0.5-s intervals) exclusively from the patient cluster, extracting 200×200 pixel windows centered on the average face midpoint for each clip. This procedure generated 500,000 short video clips (0.5s each), with each clip focused exclusively on the patient’s facial expressions. All clips were encoded in MP4 format using H.264 compression [33]. This unlabeled video corpus provides the foundation for subsequent human annotation procedures.

A detailed account of a very similar data creation and preprocessing pipeline is provided in our related preprint [34]. However, it should be noted that the dataset described there is not identical to the one used in the present study, as the preprint employs categorical emotion annotations, whereas the current dataset is annotated with free-text labels.

### 2.3 Human annotation

To generate emotion labels for each video clip, we recruited ten psychology master’s students (all native German speakers; six females, four males, age range 24–32 years, mean age = 27.6, SD=2.8). All participants held bachelor’s degrees in psychology and were recruited through university course announcements. Participants received course credit for their involvement.

Annotation was conducted using a custom-developed tool. Each annotator created an account and was presented with a randomized sequence of clips sampled without replacement. Annotators viewed clips sequentially and provided free-text descriptions of observed emotions. The specific instruction was: “Describe the emotional state of the person in this clip as if you were explaining it to a friend who cannot see the clip.”

To minimize annotation fatigue and maintain data quality, each labeling session was limited to three hours with mandatory 15-min breaks every 45 min. Each annotator contributed approximately 20 h of annotations across multiple sessions.

### 2.4 Embedding generation

The resulting dataset of video clips with emotion annotations represents our authentic emotion corpus. All free-text labels underwent preprocessing to lowercase format. Additionally, an automated spelling correction was applied to all labels to further standardize the text. Labels that were missing or marked as errors by annotators (e.g., video display issues) were designated as missing data.

Each resulting valid label was converted into a 768-dimensional, $L_{2}\text{-normalized}$ embedding using the fine-tuned German-language Sentence-BERT model german-roberta-sentence-transformer-v2. This model has been specifically fine-tuned for semantic similarity tasks [35, 36]. These embeddings represent continuous, nuanced emotional meanings for each video clip, subsequently serving as training targets for the neural network.

### 2.5 Video preparation for network training

Using the annotated video clips, we performed several processing steps to convert video data into compact, analysis-ready feature vectors. Each 15-frame clip (initially 200×200 pixels per frame, yielding 200×200×15=600,000 raw values) was processed through MediaPipe’s Face Mesh model to detect 478 three-dimensional facial landmarks (x,y and z) on the patient’s face in each frame. These landmarks correspond to anatomical reference points including the nose tip, mouth corners, and eyebrow positions.

With three coordinates per landmark point, the complete landmark set for all 15 frames is represented by 478×3×15=21,510 numerical features per clip, achieving a 96.4% dimensionality reduction from raw pixel values of the clips.

To ensure scale and translation invariance, we normalized each clip’s landmarks through a two-step process. First, landmarks were centered by subtracting the mean coordinate across all landmarks and frames within each clip. Second, the centered landmarks were scaled by the maximum inter-landmark distance within that clip. This normalization preserves relative facial movement patterns while removing variability due to camera zoom or head positioning.

The final feature representations were stored separately for each video clip using systematic file naming conventions (e.g., clip12345.csv) that reference corresponding annotations in the label database, ensuring unambiguous mapping between feature files and emotion labels.

### 2.6 Model training

We implemented and trained three distinct neural architectures using PyTorch: a fully connected deep neural network (DNN), a bidirectional long short-term memory network (BiLSTM), and a transformer encoder [37]. Each architecture was designed to map normalized facial landmark feature vectors to 768-dimensional emotion embeddings. Since all three architectures achieved comparable validation performance, we concentrated subsequent optimization and analysis on the transformer model due to its superior attention mechanisms for sequential data.

Hyperparameter optimization was conducted using Bayesian optimization using the Ax library [38]. This approach enabled efficient exploration of high-dimensional parameter spaces, including model depth, feed-forward dimensionality, attention head count, dropout rates, learning rates, and optimizer selection while minimizing computational expense.

We systematically applied Bayesian optimization across all architectures, automatically converting optimizer proposals into specific parameter values and instantiating the corresponding networks. To prevent overfitting, we employed a training, validation, and test split throughout our training. After identifying optimal hyperparameters, we conducted extended training phases, ensuring comparability across runs.

Finally, we versioned and saved learned model weights while compiling detailed results tables capturing model type, validation loss, final training loss, and chosen hyperparameters for transparent downstream analysis.

### 2.7 Model testing

Model evaluation presents unique challenges due to the absence of definitive ground truth emotions. Since our dataset relies on free-text annotations converted to continuous embeddings, we operate within semantic space rather than against discrete labels. Two annotators may describe identical video clips using different terminology, yet both descriptions may lie equally close to theoretical ideal emotional representations. Consequently, traditional metrics such as balanced accuracy or standard hypothesis testing are not directly applicable.

Instead, we demonstrate both internal and external validity of our emotion representations through complementary evaluation approaches.

#### 2.7.1 Internal validity

Internal validity assessment evaluates whether model predictions respect semantic relationships inherent in our dataset. Rather than comparing against single “true” embeddings, we compare predicted embeddings against distributions of human-annotated embeddings within the same semantic space.

For each video clip, we performed leave-one-out cross-validation: we trained our model on all clips except one, then predicted the embedding for the held-out clip. We calculated the cosine similarity between the predicted embedding and the true embedding of the held-out clip. To contextualize this value, we also computed cosine similarities between the true embedding and the embeddings of all other clips, creating a distribution of pairwise similarities. We then z-scored the predicted-vs.-true similarity relative to this distribution, so that a z-score of zero indicates prediction accuracy equivalent to randomly selecting from other embeddings. After repeating this process for every clip, we visualized the resulting z-scores to assess model performance across all clips.

#### 2.7.2 External validity

External validity was assessed by applying our trained model to standard emotion recognition datasets, treating published labels as reference standards. These labels typically serve as performance instructions for actors portraying specific emotions. For each external corpus, we applied identical preprocessing and embedding pipelines used in our primary dataset to obtain continuous label embeddings.

We executed our trained model on each external video clip to generate predicted embeddings. As reference points, we computed embeddings for corpus labels (e.g., “fear,” “joy”). Cosine similarities between video clip embeddings and label embeddings indicate the degree to which our model recognizes clip emotions as represented by target terms. These similarities were normalized using baseline distributions of human-to-human label similarities within each dataset, resulting in a comparable scale.

### 2.8 Embedding interpretation and translation to human-readable form

To make high-dimensional emotion vectors understandable to humans, we use a back-translation approach based on semantic similarity. The key idea is that both the emotion predictions from video clips and the textual descriptors are represented as 768-dimensional embeddings in the same vector space. Users can provide any emotion label they wish, ranging from single emotion words (like “joy” or “anxiety”) to short phrases (such as “feeling under pressure”), to probe the model’s internal representations.

Each emotion label is encoded using the same Sentence-BERT model as our annotation pipeline, ensuring that both clip and label embeddings are directly comparable within a shared, L${}_{2}$-normalized space. We then compute the cosine similarity between the emotion label embedding and the predicted embedding. This results in a similarity score between -1 and 1. To make these scores easier to compare across different labels and clips, we rescale them to the interval based on all used clips.

Rather than limiting interpretation to only the top-scoring labels, which would miss the nuanced and distributed nature of affective meaning, we suggest to look at the entire affinity profile for each clip. By showing ranked subsets of descriptors, users can see not only the best matches but also how related emotional concepts cluster together. This open-ended, non-categorical approach avoids the distortions that can occur in complex semantic spaces and provides a richer, more nuanced view of the model’s internal affective representations.

For visualization, we use radar charts [39], arranging descriptors in a circle. To enhance interpretability, we optimize the layout so that semantically similar labels are placed next to each other. Specifically, we treat the arrangement as a traveling salesman problem over the cosine similarity matrix of the labels, using a nearest-neighbor heuristic followed by 2-opt refinement to maximize adjacency similarity. We then plot the normalized similarity values around the circle, creating radar charts where peaks indicate the strongest affinities and smooth transitions reflect gradual semantic shifts. For each video clip, these radar charts provide an intuitive, human-readable snapshot of the model’s affective embedding, translated back into familiar language.

The complete model training pipeline and results presented in this article can be replicated using code available at https://github.com/HannesDiemerling/AFFECT. Note that clinical datasets cannot be shared openly due to privacy constraints; however, trained models remain available for research use.

### 2.9 Description of the AFFECT tool

Building on the methodology outlined above, we encapsulated our complete pipeline—from facial landmark extraction and embedding prediction to z-score computation and radar chart visualization—into a single, user-friendly application. AFFECT (Automated Fast Facial Emotion Coding Tool) translates our methodological framework into a deployable solution requiring minimal configuration. The tool supports batch processing, enabling seamless integration into automated workflows and third-party applications.

AFFECT provides fully automated pipelines for continuous emotion analysis in video data. The interface allows users to select individual video files or entire folders for analysis. Once videos are selected, the software segments each video into 15-frame windows with user-defined step lengths for sliding window analysis across complete footage. For each segment, AFFECT performs facial landmark detection, applies transformer models to predict 768-dimensional emotion embeddings, and records results in timestamped CSV files.

Disk storage requirements scale with total video duration and generated segment counts, so users processing large batches should allocate additional disk capacity proportional to footage length (Table 1). While GPUs are not strictly necessary and complete pipelines operate on multi-core CPUs, leveraging high-performance GPUs with CUDA support (e.g., NVIDIA GTX 10 series or newer) can accelerate processing by orders of magnitude. AMD or Intel GPUs require manual adaptation of CUDA-based code components. Peak memory usage depends on input data characteristics, with 16 GB sufficient for moderate workloads and standard video files, while 32 GB or more is recommended for high-throughput or parallelized analyses.

**Table 1:** System requirements for AFFECT.

Requirement	Minimum	Recommended
CPU	Intel Core i5-8600K or equivalent	AMD Ryzen 7 5800X or equivalent
RAM	16 GB	32 GB
GPU	NVIDIA GTX 1050 Ti (optional)	NVIDIA RTX 3060 or better
Storage	10 GB SSD	100 GB SSD

Users specify emotion anchor term sets for representing recognized emotions. These might include basic emotion sets, dimensional model labels, or application-specific descriptions (e.g., psychotherapeutic contexts). For each video segment, AFFECT calculates cosine similarities between emotions and anchor terms as z-scores relative to entire batches. Results are stored in CSV format for compatibility with standard software. Optional radar chart rendering produces image files for each segment, and composite video generation displays original footage alongside corresponding radar charts. AFFECT is distributed as Python source code and standalone executables for Windows and Linux, facilitating both interactive use and pipeline integration.

Source code and the AFFECT application are available on GitHub at https://github.com/HannesDiemerling/AFFECT.

## 3 Results

This section presents the primary outcomes of our study, beginning with descriptive statistics characterizing both the human annotation process and patient video corpus, followed by internal and external validity analyses, and concluding with interpretability examples demonstrating how AFFECT’s embeddings translate into human-readable emotion profiles.

### 3.1 Descriptive statistics

We first summarize key properties of our dataset and annotation procedures.

#### 3.1.1 Labeler demographics

Table 2 summarizes the demographic characteristics of our ten annotators. They ranged in age from 24 to 32 years (M=27.6, SD=2.8), included 6 females and 4 males, and held a bachelor’s degree in psychology. All participants were native German speakers and received course credit for their participation.

**Table 2:** Annotator demographics (N=10).

Metric	Age	Gender	Highest degree
Mean (SD)	27.6 (2.8)	6 F, 4 M	10 B.Sc.
Range	24–32	–	–

#### 3.1.2 Patient video characteristics

Table 3 reports the key attributes of the patient video corpus. We collected N=500,000 clips from 20 unique patients (12 females, 8 males), aged 22–64 (M=41.2, SD=12.7). Clinical diagnoses spanned mood, anxiety, and personality disorders, with the most prevalent being major depressive disorder (30%), generalized anxiety disorder (25%), personality disorders (15%), and other conditions (30%).

**Table 3:** Patient video corpus characteristics.

Attribute	Value
Number of clips	500,000
Number of patients	20
Patient age, Mean (SD)	41.2 (12.7) years
Gender distribution	12 female, 8 male
Primary diagnoses	MDD (30%), GAD (25%), PD (15%), Others (30%)

MDD, major depressive disorder; GAD, generalized anxiety disorder; PD, personality disorder.

#### 3.1.3 Generated label characteristics

Table 4 presents statistics on the collected free-text labels. Annotators produced an average of 8.2 words per clip (SD=1.9), generating 4,100 unique labeled clips across the entire corpus. Each annotator labeled on average 734 clips (SD=341.94). The mean pairwise cosine similarity among label embeddings was 0.5 (SD=0.12) when computed within individual annotators and 0.3 overall (range: 0.05–0.75), indicating moderate semantic diversity across the dataset.

**Table 4:** Label annotation statistics.

Metric	Mean (SD)	Range	Notes
Words per label	8.2 (1.9)	3–15	Average words per clip description
Unique labels (total)	4,100	–	Across entire corpus
Clips per annotator	734 (341)	–	Average per individual
Cosine similarity (within-person)	0.5 (0.12)	–	Mean pairwise similarity
Cosine similarity (overall)	0.3	0.05–0.75	Across all annotations

### 3.2 Internal validity

**Figure 1:**
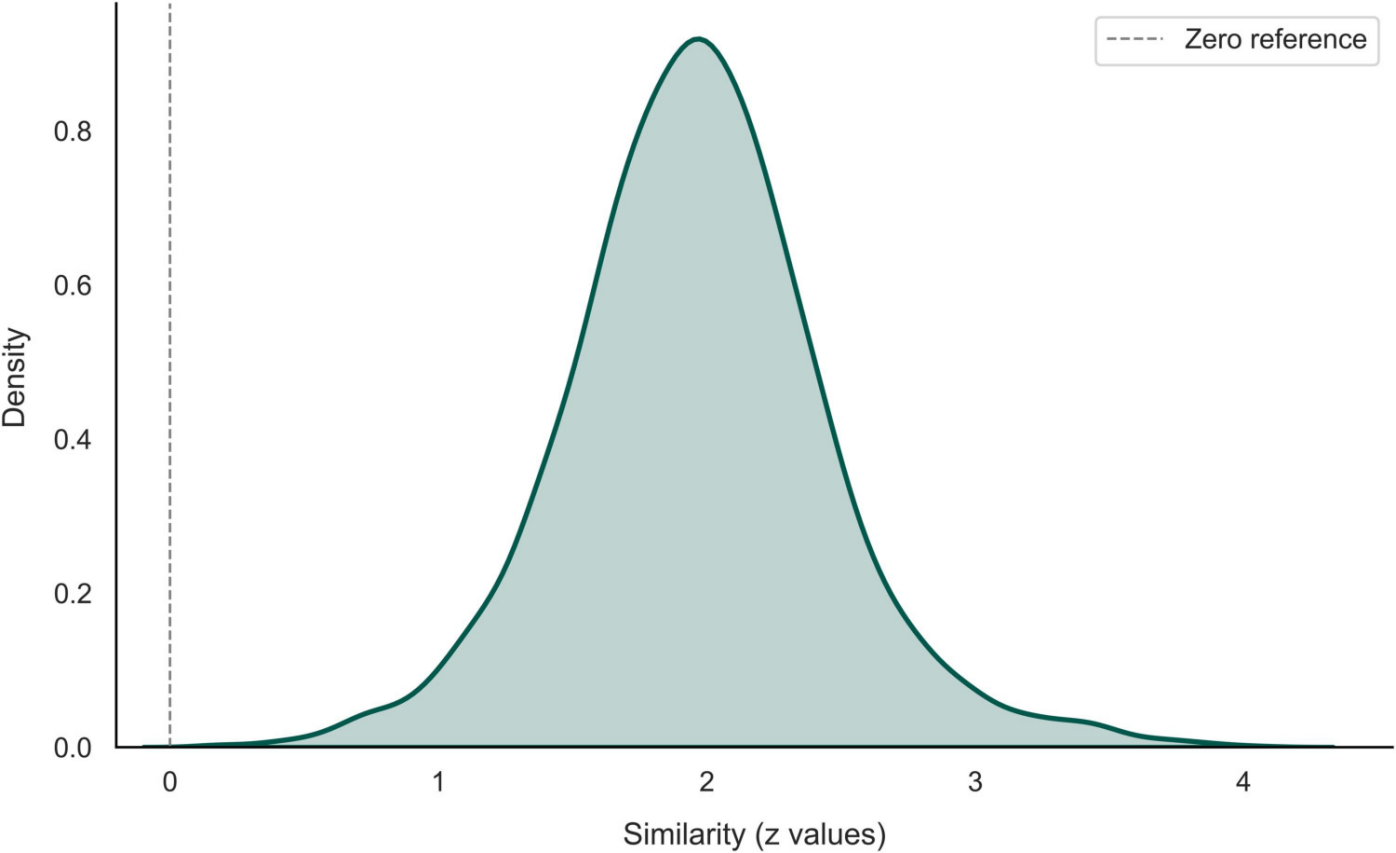
Standardized cosine similarity (z-scores) distribution comparing model predictions with their corresponding true labels. The distribution’s positive shift provides evidence for the internal validity of our emotion recognition model.

Figure 1 displays the standardized z-score distribution for cosine similarities between model predictions and their corresponding ground-truth labels. These z-scores were calculated by comparing each prediction-truth similarity against the distribution of similarities between that prediction and all other possible labels in the dataset. The consistently positive z-scores demonstrate a mean value of 1.97 standard deviations above zero ($CI_{95\%}=[1.95,1.98]$), indicating that model predictions are significantly more similar to their true labels than would be expected by random selection from the label distribution.

### 3.3 External validity

**Figure 2:**
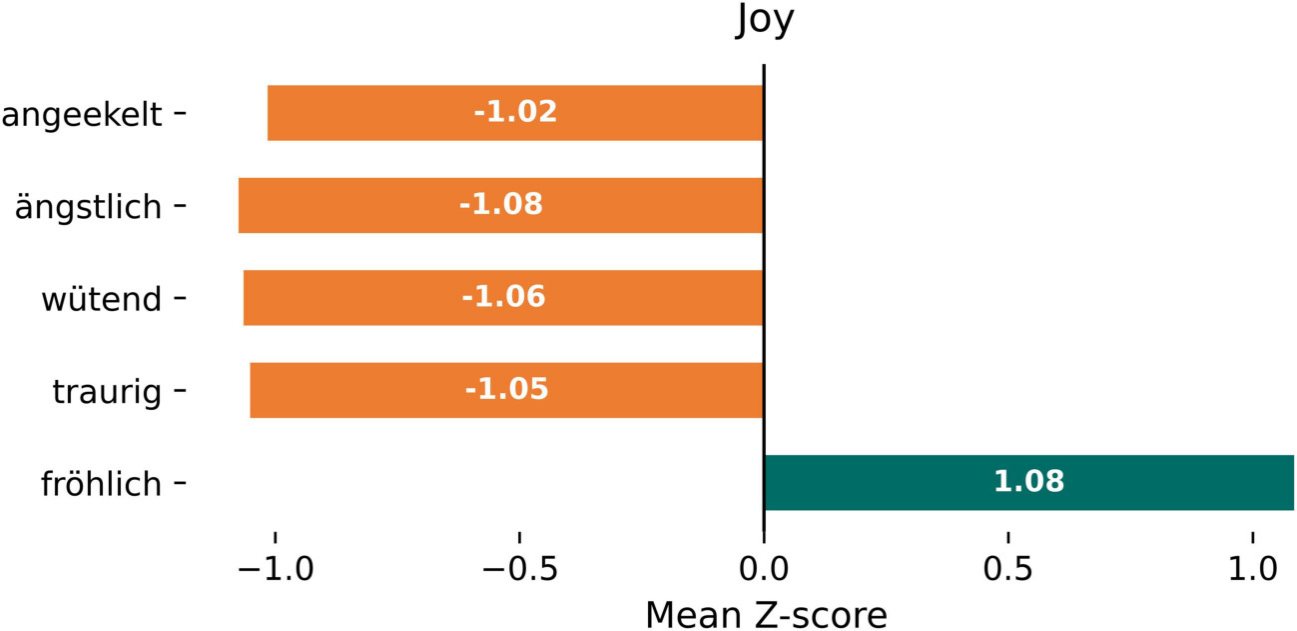
Diverging bar chart of mean standardized cosine similarity (z-scores) between predicted embeddings and each basic emotion label for clips truly labeled “happy” (RAVDESS). Bars extending rightward indicate above-baseline similarity.

**Figure 3:**
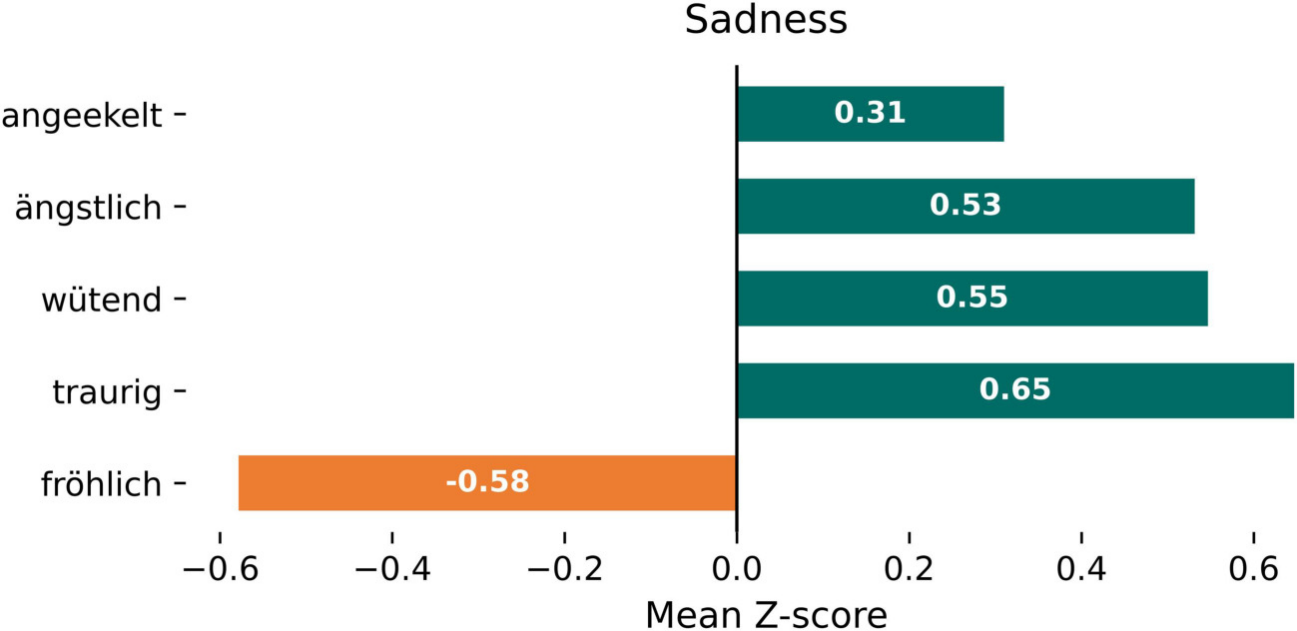
Diverging bar chart of mean standardized cosine similarity (z-scores) for clips labeled “sad” (RAVDESS).

**Figure 4:**
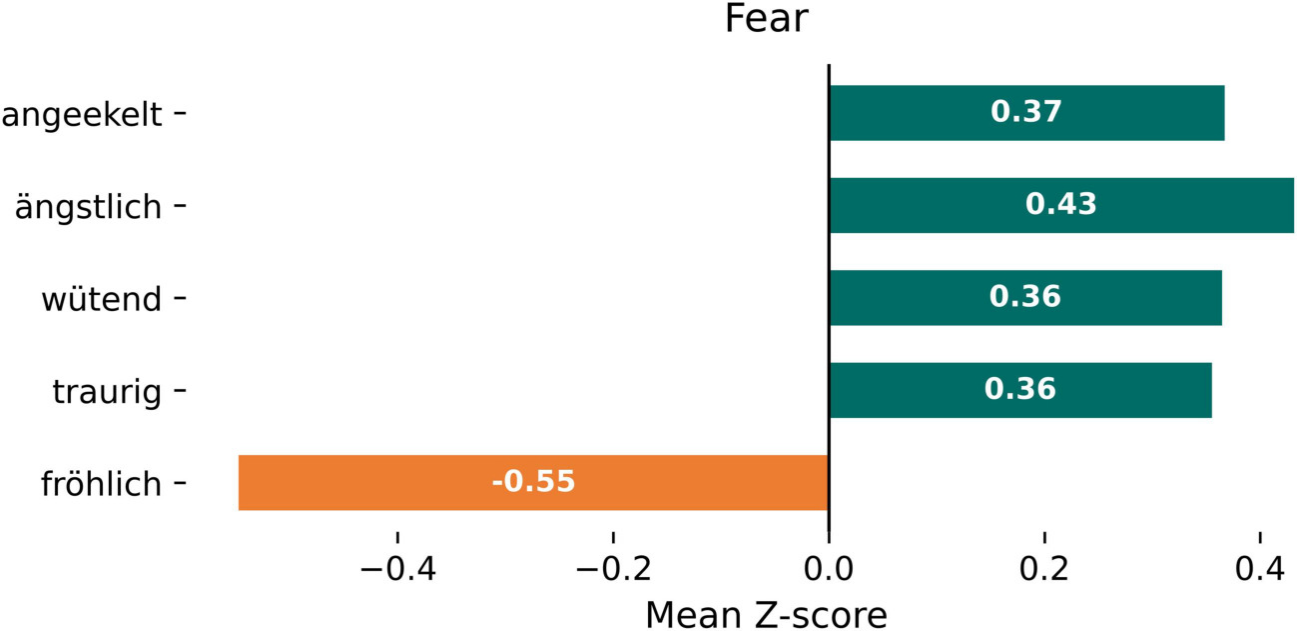
Diverging bar chart of mean standardized cosine similarity (z-scores) for clips labeled “fearful” (RAVDESS).

**Figure 5:**
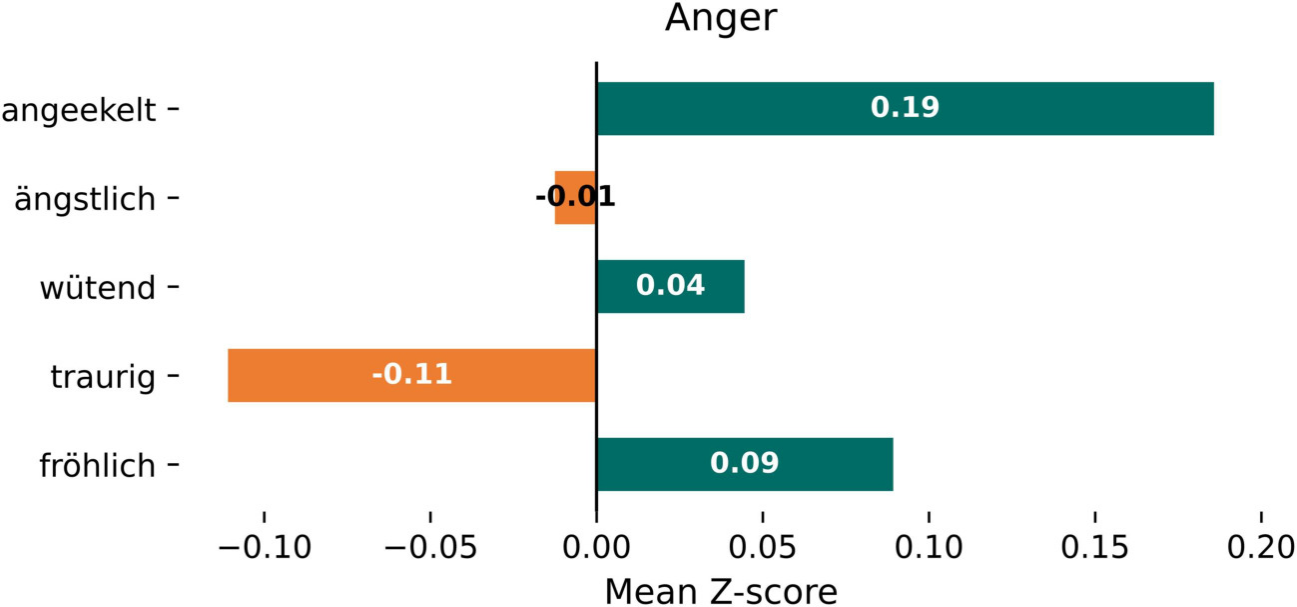
Diverging bar chart of mean standardized cosine similarity (z-scores) for clips labeled “angry” (RAVDESS).

**Figure 6:**
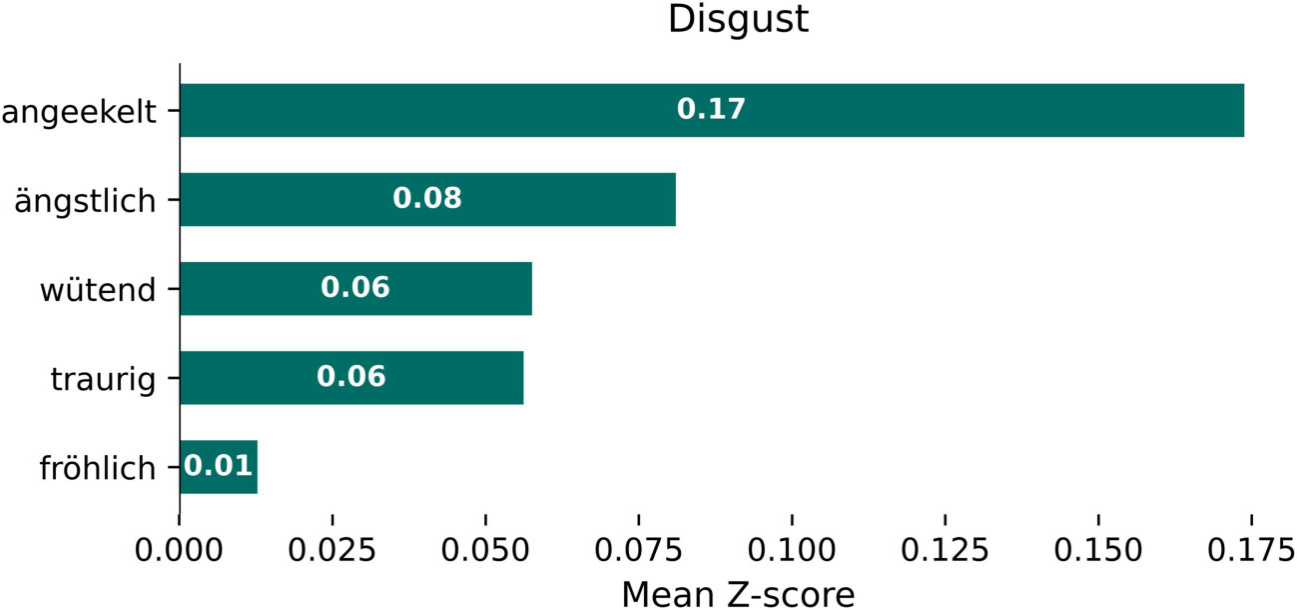
Diverging bar chart of mean standardized cosine similarity (z-scores) for clips labeled “disgusted” (RAVDESS).

We evaluated our transformer model on the standard acted-emotion corpora RAVDESS [27], computing z-scored cosine similarities between predicted video clip embeddings and embeddings of the published emotion labels. The diverging bar charts in Figures 2– 6 show mean standardized cosine similarities across all video clips labeled with each basic emotion.

Four of the five basic emotions (joy, sadness, fear, and disgust) demonstrated highest affinity scores corresponding to their true categories. For joy, model embeddings were significantly closer (mean z=1.08, SD=0.82) to the “joy” descriptor than to any other basic emotion (Figure 2). Similar patterns emerged for sadness (mean z=0.65, SD=0.95; Figure 3), fear (mean z=0.43, SD=0.71; Figure 4), and disgust (mean z=0.17, SD=0.75; Figure 6).

The exception was anger (mean z=0.04, SD=0.87; Figure 5), where video clips were typically closer to disgust than to other basic emotions, likely reflecting the subtle nature of anger expressions in clinical contexts compared to the high-arousal, exaggerated anger portrayals typical in acted datasets.

### 3.4 Combined interpretability results

**Figure 7:**
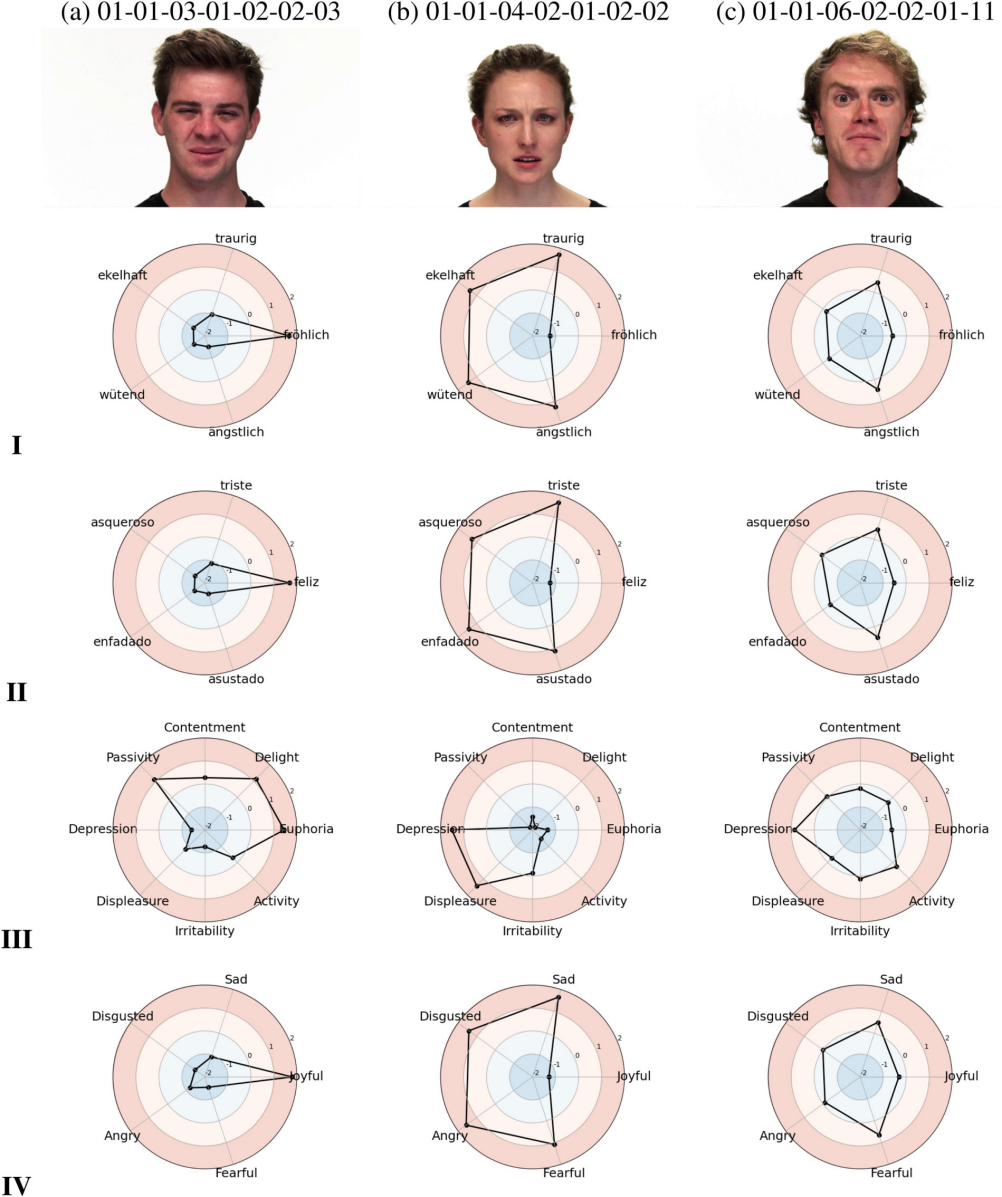
Presentation of emotion embeddings for three video clips using different verbal representations. Columns represent individual video clips; rows show radar plots depicting emotion alignment with respective anchor sets: basic emotions in German (I) and Spanish (II), Russell’s extended circumplex model (III), and basic emotions in English (IV). Portraits in panels (a), (b), and (c) are screenshots from videos from 01-01-09-01-02-02-03, 01-01-04-02-01-02-02 and 01-01-06-02-02-01-11 by Steven R. Livingstone and Frank A. Russo, licensed under CC BY-NC-SA 4.0.

The high-dimensional emotion embedding space enables flexible translation back into human-accessible verbalizations. Figure 7 demonstrates example presentations of three emotion videos from the RAVDESS database, where actors were instructed to portray joy (a), fear (b), and sadness (c).

Row I displays radar plots depicting predicted emotion similarity to the five basic emotions in German, the language of the dataset. For all three clips, the expected emotion demonstrates the strongest affinity, although minor contributions from other basic emotions are also present, reflecting the nuanced nature of our continuous representation.

Row II presents radar plots illustrating the predicted emotional similarities to the five basic emotions in Spanish. As shown, the patterns across languages are highly similar, demonstrating the predictions’ multilingual capabilities.

Row III visualizes emotions using an extended Russell’s circumplex model, with valence and arousal dimensions arranged orthogonally and representative emotions positioned within each quadrant. Each clip appears in its theoretically expected quadrant, and all plots exhibit convex shapes, indicating that quadrant representatives effectively capture the dimensional composition of the emotions.

Row IV employs English translations of the basic emotions. The radar plots differ slightly, but rows I, II, and IV are nearly indistinguishable. This demonstrates that our emotion embedding model transfers effectively to basic emotion frameworks, at least for the tested Indo-European languages.

Importantly, all four visualizations per clip (per column) originate from the same predicted embedding vector, differing only in how this vector is mapped onto linguistic, dimensional, or clinical interpretative frameworks.

## 4 Discussion

This study created and leveraged a new corpus of authentic emotion expressions recorded during naturalistic psychotherapy sessions, annotated with natural language descriptions of observed emotions. Building on this resource, we developed a high-dimensional emotion embedding representation and introduced AFFECT, a comprehensive end-to-end tool for continuous automated emotion recognition in video data.

Our dataset represents a fundamental departure from benchmark collections such as RAVDESS and EMO-DB, which achieve exceptional production quality through controlled, high-fidelity portrayals by trained actors but may not capture the spontaneity and subtlety characteristic of authentic emotional exchanges [27, 28]. In contrast, our corpus captures genuine therapist–patient interactions featuring natural, context-rich affective signals that reflect real-world human emotion expression. This ecological validity necessarily involves trade-offs, including reduced experimental control and a participant pool limited to clinical populations rather than broader normative samples.

Our annotation procedure leverages the descriptive capabilities of trained human evaluators, who provided free-text emotion labels rather than selecting from predefined categorical schemes. The instruction to “describe the emotional state as if explaining it to a friend” results in more complex and potentially nuanced descriptions that capture emotional blends and gradations [6, 13]. This approach reflects our fundamental assumption that emotions cannot be adequately characterized by narrowly defined theoretical models, which inevitably oversimplify the complexity of human affective experience. Instead, high-dimensional embedding spaces provide largely unconstrained, dense representations of emotional information, with established emotion theories serving as interpretive lenses that highlight different patterns at varying levels of granularity.

Our emotion representation employs 768-dimensional Sentence-BERT embeddings [36], encoding the informational content of each annotation as coordinates in continuous semantic space. This approach preserves fine-grained distinctions while enabling systematic comparisons across descriptions. However, embedding spaces vary with pre-trained model selection: different architectures, training corpora, or language variants may yield different affective geometries, potentially influencing downstream tool behavior when computing similarities to human-readable emotion descriptors.

The embedding space coordinates for each video reflect the distribution of human annotations, rather than representing single “correct” labels. Our model is trained to map video features into semantic regions that align with these annotation distributions. To evaluate performance, we calculate the cosine similarity between the predicted embeddings and the distributions derived from human annotations, acknowledging that neither individual human labels nor model outputs serve as absolute ground truth.

Internal validation using leave-one-out cross-validation revealed that model-predicted embeddings consistently fall much closer to held-out human annotations than to baseline human–human similarity distributions. This indicates that our model captures latent semantic structures of affective meaning rather than overfitting to specific annotation instances [19].

External validation comparing AFFECT’s outputs against the acted dataset RAVDESS [27] yielded strong alignment for joy, sadness, fear, and moderate alignment for disgust, while agreement for anger remained limited. This pattern likely reflects fundamental differences between high-arousal, exaggerated expressions typical in acted datasets—such as intense disgust or aggressive outbursts—and the more subtle emotional displays characteristic of clinical interactions [2]. Training on therapy videos provides particular value in naturalistic contexts and could also yield promising results when applied to highly stylized emotional portrayals, although some differences in generalizability are expected and need further study.

To enhance interpretability, AFFECT generates similarity measures between high-dimensional embeddings and user-specified anchor terms that facilitate emotion description. Radar charts visualize emotional positioning relative to these descriptors (Figure 7), with this approach’s flexibility allowing users to compare self-selected term lists across languages or theoretical frameworks while visualizing graded similarities to each term. This model-independent visualization underscores how our continuous representation serves as a common substrate onto which various emotion theories act as interpretive “lenses”, each selecting and highlighting different aspects of identical underlying affective information [40].

AFFECT itself is distributed as an open-source tool (Apache 2.0 license) available via GitHub. In its current implementation, users provide video material featuring a single person’s face, after which AFFECT automates face detection, landmark extraction, transformer-based embedding prediction, and radar-chart generation. Sliding-window analysis using 15-frame windows with adjustable step sizes produces time series of emotion profiles, enabling detailed, continuous tracking of affective dynamics. This end-to-end pipeline embodies our vision of a comprehensive solution for nuanced emotion analysis across research and applied contexts.

### 4.1 Limitations and future directions

While our dataset’s authenticity represents a major strength, it remains constrained to a German clinical outpatient population, potentially limiting generalizability to healthy or demographically diverse groups. The free-text annotation procedure, though informative, introduces variability based on individual annotator styles; future research could explore semi-structured prompting approaches or incorporate multiple embedding architectures to assess robustness. Our reliance on German-language fine-tuned embedding models means that affective geometries could shift under alternative pre-trained representations, suggesting systematic comparison of embedding variants as a priority for future investigation. Additionally, AFFECT currently processes only video input; integrating multimodal signals including audio or physiological data will further enhance emotion recognition capabilities.

Future research should focus on several critical directions to further strengthen and extend the current approach. Expanding data collection to include not only patient speech but also therapist responses, as well as interactions in non-clinical contexts such as everyday conversations, group settings, and virtual environments, will be essential for evaluating the broader applicability of AFFECT. Integrating naturalistic therapy recordings with carefully selected material from acted corpora such as RAVDESS and CREMA-D can help address current limitations in emotional intensity and improve the representation of rare affective states. In addition, systematic comparisons of embedding architectures, including multilingual and monolingual transformers, distilled and full-scale models, and non-transformer alternatives, should be conducted to assess semantic bias, robustness to paraphrasing, and transferability across domains. Progress toward comprehensive multimodal fusion is also necessary; this involves synchronizing facial landmark data with speech prosody, linguistic transcripts, and physiological signals such as EKG, EEG, skin conductance, and eye tracking. It is important to investigate whether audio and visual features can predict missing physiological information in order to enable reliable emotion estimation in environments with limited sensor data. Finally, developing AFFECT as a real-time, user-friendly platform that is accessible through web APIs, desktop interfaces, or mobile applications, and that supports modular integration of multiple languages, theoretical models such as the circumplex and appraisal frameworks, and diverse application domains including therapy, education, and marketing, will be crucial for practical deployment. Together, these improvements will enable more accurate, flexible, and context-sensitive emotion recognition in both research and applied settings.

## 5 Conclusion

This work introduces a new approach to automated emotion recognition based on continuous semantic embeddings from free-text emotion descriptions in authentic psychotherapy sessions. By training transformer models on a dataset of over 6,000 labeled clinical video clips, we demonstrate that high-dimensional embeddings can capture the complexity of human emotions. Our method allows flexible interpretation using different theoretical frameworks and supports analysis with arbitrary emotion descriptors.

To enhance interpretability, AFFECT computes similarities between emotion embeddings and user-chosen anchor terms, visualized as radar charts. This model-independent approach allows any emotion theory or term list to act as an interpretive “lens,” highlighting different aspects of the same underlying affective information.

The AFFECT tool implements this approach in a fully automated, user-friendly pipeline, enabling high-resolution, sliding-window emotion analysis and clear visualizations. Internal and external validations confirm that our system reliably detects key emotions such as joy, sadness, and fear, and aligns well with established emotion categories.

Our findings suggest that continuous, data-driven representations offer clear advantages for emotion recognition, supporting both research and clinical applications. Future work will extend the dataset to more diverse populations, compare alternative embedding models, and integrate multimodal signals for richer emotion analysis. The open-source release of AFFECT makes these advances broadly accessible, laying the groundwork for improved emotion monitoring, feedback, and intervention in real-world settings.

## Data Availability

The raw data supporting the conclusions of this article will be made available by the authors, without undue reservation, subject to the formal approval of the data owners and the relevant institutional review boards.
